# Neuroendocrine Neoplasms of the Gallbladder: A Clinicopathological Analysis of 13 Patients and a Review of the Literature

**DOI:** 10.1155/2021/5592525

**Published:** 2021-05-22

**Authors:** Pengyan Wang, Jingci Chen, Ying Jiang, Congwei Jia, Junyi Pang, Shan Wang, Xiaoyan Chang

**Affiliations:** Department of Pathology, Peking Union Medical College Hospital, Chinese Academy of Medical Sciences and Peking Union Medical College, Tsinghua University, Beijing 100730, China

## Abstract

**Objectives:**

Mixed neuroendocrine–non-neuroendocrine neoplasms (MiNENs) are rare gallbladder neuroendocrine neoplasms (GB-NENs). This study is aimed at investigating the clinicopathological features of GB-NENs and identifying prognostic factors related to overall survival (OS) of GB-MiNENs.

**Methods:**

The clinical data and pathological features of 13 patients with GB-NENs in our hospital were retrospectively reviewed. Additionally, 41 GB-MiNENs cases reported in English literature were reviewed and survival analysis was performed.

**Results:**

The mean age of thirteen patients (6 males and 7 females) with GB-NENs was 57.2 years (range: 35-75 years). Two patients were diagnosed with NET grade 1 (G1), two patients with NEC (large cell/small cell = 1/1), and nine patients with MiNENs. Of these 9 patients with MiNENs, 8 had composite tumors and 1 had amphicrine carcinoma. Microscopically, the adenocarcinoma component was located in the surface mucosa, and the neuroendocrine component was in the area of deep invasion, liver infiltration, and lymph node metastasis. Total analysis of 41 GB-MiNENs showed that patients were mainly elderly women (female/male ratio, 2.4 : 1.0; median age, 60 years). Kaplan-Meier's analysis demonstrated that liver metastasis and TNM stage III-IV were associated with decreased OS (*P* < 0.05), whereas age, sex, tumor size, grade of the neuroendocrine component, lymph node metastasis, and adjuvant chemotherapy were not significantly prognostic indicators of OS. Multivariate analysis identified liver metastasis (hazard ratio = 4.262, 95%confidence interval = 1.066‐17.044, *P* = 0.040) as an independent unfavorable prognostic factor.

**Conclusions:**

GB-MiNENs were the most common type of GB-NENs in our case series, and neuroendocrine components exhibited more aggressive lymph node metastasis and local invasion than adenocarcinoma. Liver metastasis was a poor prognostic indicator in GB-MiNENs patients.

## 1. Introduction

Neuroendocrine neoplasms (NENs) originate from disseminated neuroendocrine cells and account for approximately 0.5% of all newly diagnosed malignancies [1]. Most of these tumors are found in the gastrointestinal and respiratory systems, accounting for 66% and 31%, respectively [2]. The gallbladder mucosa is absent from neuroendocrine cells except for those in the gallbladder neck region [3, 4], which is why NENs of the gallbladder are rare and account for only 0.5% of all NENs and 2% of all gallbladder tumors [5].

According to the World Health Organization (WHO) 2019 classification, gallbladder neuroendocrine neoplasms (GB-NENs) are divided into neuroendocrine tumors (grade 1, 2, or 3 NETs), neuroendocrine carcinomas (NECs, large cell or small cell type), and mixed neuroendocrine–non-neuroendocrine neoplasms (MiNENs) [6]. Mixed adenoneuroendocrine carcinomas (MANECs) were renamed MiNENs in the WHO 2017 classification [7]. MiNENs are neoplasms in which areas of the neuroendocrine component intermingle with areas of the non-neuroendocrine component, each comprising at least 30% of the tumor [6]. In 1987, Lewin was the first to propose three combinations of this unique type of tumor: collision, composite, and amphicrine [8].

To date, only a few primary GB-NENs have been reported in the English language literature. The clinicopathological characteristics and prognoses for NENs of the gallbladder remain largely undetermined. Therefore, we present the pathological and clinical features of a series of 13 patients with GB-NENs and a brief literature review.

## 2. Methods and Materials

### 2.1. Patients and Clinicopathological Characteristics

From September 2000 to December 2020, the medical records of 13 patients with GB-NENs (6 males and 7 females) were retrieved from the archive files of the Department of Pathology, Peking Union Medical College Hospital. The clinical and follow-up data were obtained from electronic medical records, from the hospital discharge summary, or through telephone inquiry. Patient data were analyzed to the last follow-up before December 1, 2020. All patients underwent a cholecystectomy to extensive surgical resections, including regional lymphadenectomy and partial liver resection. Overall survival (OS) was defined as the time from surgery to the date of death or the last follow-up. Hematoxylin and eosin (H&E) slides and immunohistochemistry (IHC) results were reviewed by three experienced pathologists. Tumor characteristics evaluated on routine H&E-stained slides included the tumor growth pattern, cell type, mitotic index (10/high-power fields (HPFs)), stroma, and necrosis. Other pathological features that were also examined included tumor size, gross classification (protruding or infiltrative), depth of invasion, and lymph node metastasis. Staging was determined according to the Union for International Cancer Control/American Joint Committee on Cancer (UICC/AJCC) 8th edition [9].

Based on histopathological features, the WHO (2019) classifies NENs into four categories: (1) NET G1 (mitotic count < 2/10 HPFs and/or Ki − 67 index < 3%), (2) NET G2 (mitotic count = 2‐20/10 HPFs and/or Ki − 67 index = 3‐20%), (3) NET G3 (mitotic count > 20/10 HPFs and/or Ki − 67 index > 20%), (4) NEC (mitotic count > 20/10 HPFs and/or Ki − 67 index > 20%), and (5) MiNENs [7]. The diagnosis of MiNENs was made based on the WHO 2019 classification, which states that each tumor component comprises at least 30% of the specimen. The combinations of the neuroendocrine and adenocarcinoma components in MiNENs were classified as follows: “collision” (the two components are clearly demarcated); “combined” (the two components are intimately and diffusely admixed); and “amphicrine” (both components are coexpressed in the same cells) [8, 10–12]. This study was approved by the Institutional Review Board of Peking Union Medical College Hospital.

### 2.2. IHC Analyses

All 13 patients included in this study were analyzed by IHC. The immunohistochemical analysis was performed on paraffin-embedded sections on a DAKO Autostainer. The primary antibodies used in the study included synaptophysin (SP11, dilution 1 : 100; Thermo Fisher Scientific), chromogranin A (DAK-A3, dilution 1 : 100; DAKO), Ki-67 (MIB-1, dilution 1 : 200; DAKO), cluster of differentiation protein 56 (123C3, dilution 1 : 100; DAKO), CK7 (OV-TL12/30, dilution 1 : 400; DAKO), CK19 (RCK 108, dilution 1 : 100, DAKO), and cytokeratin (AE1/AE3, dilution 1 : 100; DAKO). Appropriate positive and negative controls were used for all antibodies tested. For each immunohistochemical procedure, antigen retrieval was performed in a citrate buffer, and detection was amplified with the DAKO EnVision System. Mitoses were counted in at least 50 HPFs (1 HPF = 2 mm^2^), and the Ki-67 index was defined using the MIB antibody as the percentage of 500-2000 cells counted in areas of the strongest nuclear labeling (“hot spots”) [6].

### 2.3. Statistical Analyses

An English literature search was performed in December 2020 to identify all of the studies that reported gallbladder mixed neuroendocrine–non-neuroendocrine neoplasms (GB-MiNENs) [4, 13–40]. SPSS version 23.0 (SPSS Inc., Chicago, IL, USA) and GraphPad Prism version 7 (GraphPad Software, CA, USA) were used for statistical analyses. The Kaplan-Meier method was used for analysis of survival data, and differences were assessed using the log-rank test. The Cox regression analyses were employed to evaluate independent prognostic factors associated with GB-MiNENs. *P* < 0.05 was considered statistically significant.

## 3. Results

### 3.1. Clinicopathological Information

**Figure 1:**
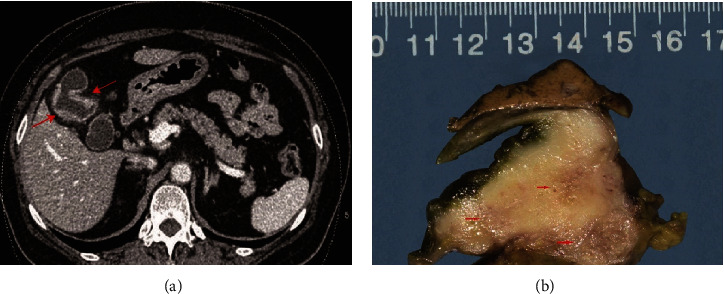
(a) An abdominal contrast-enhanced CT scan showing diffuse thickening of the gallbladder wall, with significant enhancement (arrows) in the portal venous phase. (b) Gross image of the gallbladder showing a thickened wall; the cut surface shows a tan-white thickened submucosal layer (arrows) invading through the wall into the surrounding adipose tissue, which did not invade the adjacent liver parenchyma.

Thirteen patients diagnosed with GB-NENs were evaluated in the current study. Their clinical characteristics are summarized in Table 1. Among the 13 patients, 6 were males, and 7 were females with a male-to-female ratio of 1 : 1.2. The mean age at diagnosis was 57.2 years (range 35-75 years). The imaging studies, such as ultrasound examination, contrast-enhanced computed tomography (CT) scanning, and magnetic resonance imaging (MRI), showed that eight patients had intramural protruding masses. Specifically, 5 patients had gallstones with diffuse thickening of the gallbladder wall, and 3 patients presented in an advanced stage with infiltration of the liver parenchyma (Figure 1(a)).

**Figure 2:**
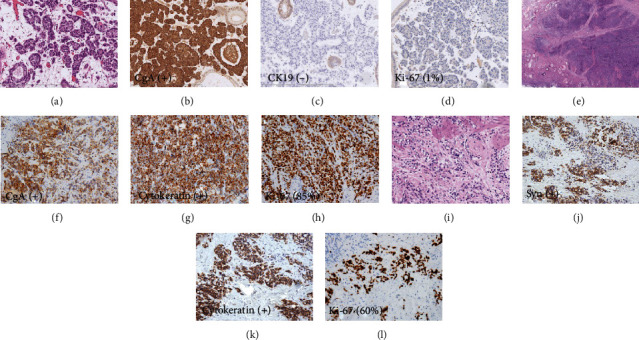
H&E staining and IHC studies of GB-NENs. (a) H&E staining of NET G1 (case 13). (b–d) IHC staining of NET G1 (case 13). (e) H&E staining of GB-MiNENs (case 2). (f–h) IHC staining of GB-MiNENs (case 2). (i) H&E staining of amphicrine carcinoma (case 9). (j–l) IHC staining of amphicrine carcinoma (case 9).

Histological features are listed in Table 2, and immunohistochemical data are provided in Table 3. Grossly, these tumors measured 0.6 to 7 cm in the greatest dimension and were gray-white to yellow with clear, identifiable boundaries (Figure 1(b)). Lesions are situated in the fundus of the gallbladder in 7 patients, in the body in 3 patients, and in the neck in 3 patients. Microscopically, two patients had NET G1, two patients had NEC (large cell/small cell = 1/1), and nine patients had MiNENs (Figure 2).

Of these 9 patients with MiNENs, 3 (33.3%) had NEC of the small cell type (SCC), 4 (44.5%) had histomorphology of large cell NEC (LCNEC), 1 (11.1%) had amphicrine carcinoma, and 1 (11.1%) had NET G2. The neuroendocrine cells were arranged in sheets with areas of a large nest, trabecula, and cord. These cells were positive for the expression of neuroendocrine markers, such as chromogranin A, synaptophysin, and CD56, but negative for epithelial markers (cytokeratins CK7 and CK19). The glandular component was composed of tubular and papillary structures formed by columnar, goblet, and Paneth-like cells, which were positive for epithelial markers but negative for neuroendocrine markers.

The present 9 patients with GB-MiNENs were classified as follows: composite tumors (8 patients) and amphicrine carcinoma (1 patient). Of the 8 patients with composite tumors, 5 patients had mainly neuroendocrine components, and 3 patients had adenocarcinoma components. The two components were closely intermingled and difficult to separate in most of the lesions. Both components invaded through the adventitia with the deeper infiltrating tumor exhibiting strong expression of the neuroendocrine markers chromogranin and synaptophysin. In two of the eight patients (patients 4 and 6), the neuroendocrine carcinoma had directly invaded into liver parenchyma. Furthermore, the neuroendocrine components of patients 4 and 1 were involved in lymph node metastasis. There was 1 amphicrine carcinoma (patient 9) that was predominantly composed of nests of cells with moderate atypia, finely and spotted nuclei, and focal mucin lakes, which exhibited concurrent neuroendocrine and nonendocrine differentiation (Figure 2).

### 3.2. Immunohistochemical Analysis

Immunohistochemistry for GB-NENs is shown in Table 3. Immunohistochemical examinations showed positivity rates of 100% for synaptophysin, 92.3% for chromogranin A, and 66.7% for CD56. All the patients showed positive Ki-67 staining by IHC with a range of 1% to 85%. In addition, almost every amphicrine carcinoma tumor cell (patient 9) showed diffuse and strong expression of synaptophysin and chromogranin A and low immunoreactivity of cytokeratin. The Ki-67 labeling index was 60%.

### 3.3. Treatment Outcomes

Follow-up was available in 13 patients with a median survival of 11.5 months (3-40 months) (Table 1). Six patients received adjuvant chemotherapy, and 4 patients refused treatment. Two patients with NET G1 (patients 12 and 13) had no recurrence during 213-month and 6-month follow-up periods, respectively, after cholecystectomy without adjuvant therapy. Patient 7 underwent radical cholecystectomy and received six cycles of chemotherapy using cisplatin and etoposide; this patient exhibited disease-free survival (DFS) after a 54-month follow-up period. The remaining patients died of tumor progression with or without adjuvant treatment; however, the patient with amphicrine carcinoma did not undergo chemotherapy and died due to recurrence 40 months after surgery, which was much longer than the median survival time of patients with GB-MiNENs (11.5 months).

### 3.4. Total Analysis with Cases Reported in the Literature

We reviewed the clinical presentation and management of 41 patients with MiNENs of the gallbladder in the published literature as well as 9 patients from our institute, as shown in Table 4 [4, 13–40]. According to our data, GB-MiNENs were more frequent in females than males (female/male ratio, 2.4 : 1.0), and the median age at presentation was 60.0 years (range 34 to 85 years). Most of the patients presented with abdominal pain (62.9%), followed by asymptomatic cases (20.0%). The tumors were commonly reported to be in the fundus (55.2%) of the gallbladder. The diameters of the tumors ranged from 1.0 cm to 15.0 cm (mean size 4.9 cm), which usually present as nodular masses (75.0%). Approximately 38.5% of the reported cases had gallstones. Nineteen of 41 (46.3%) cases were large cell type (LCNEC), which was the most common type of GB-MiNENs; however, only two (4.9%) exhibited amphicrine carcinoma in morphology and immunohistochemistry. Among the cases reporting the histology of metastases, most of the lymph node metastasis, liver infiltration, and distant metastasis were composed of the pure neuroendocrine component [14, 18, 21, 23, 29, 34, 37].

**Figure 3:**
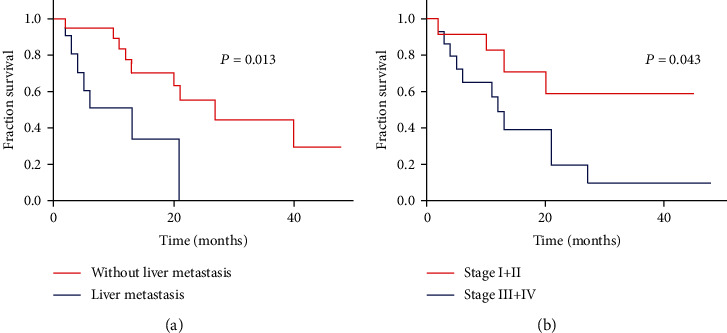
The Kaplan-Meier survival analysis shows the relationship of liver metastasis (a) and tumor stage (b) with overall survival in GB-MiNENs patients.

In the subsequent prognostic analysis, the Kaplan-Meier method was used to evaluate prognostic factors, including age, sex, tumor diameter, neuroendocrine component, lymph node metastasis, liver metastasis, TNM tumor stage, and adjuvant chemotherapy, as shown in Table 5. Only thirty patients with survival data were analyzed, with a median OS of 11 months (2-27 months). Liver metastasis and TNM tumor stage were identified as significant predictors of OS (Figure 3). Univariate and multivariate regression analyses were performed to assess the factors related to OS, and liver metastasis (hazard ratio = 4.262, 95%confidence interval = 1.066‐17.044, *P* = 0.040) was found to be independently associated with poor survival, as shown in Table 6.

## 4. Discussion

GB-NENs are rare but highly malignant gallbladder tumors, most of which are found after cholecystectomy for cholecystitis, surgery for a suspected biliary malignancy, or autopsy [41]. The current literature has shown that more than one-third of gallbladder NECs are combined with an adenocarcinoma component (MiNENs) [6]. By definition, MiNENs are regarded as a conceptual category and diagnosed only when both components are present in more than 30% of the tumor based on pathological examinations [6]. To date, only a few patients with GB-NENs have been described in the literature, and even fewer with GB-MiNENs have been described.

The pathogenesis of GB-NENs remains controversial. Some researchers believe that this malignancy originates from the gastric or intestinal metaplasia of the gallbladder epithelium, which may explain the coexistence of cholecystitis and cholelithiasis in patients with GB-NENs [42]. In our study, we observed that 5 of 13 patients had gallstones with cholecystitis. However, recent studies revealed that the different components of MiNENs have similar mutation profiles, suggesting a common monoclonal origin [39, 43, 44]. In our case series, 69.2% (9/13) of gallbladder neuroendocrine neoplasms were accompanied by adenocarcinoma components, suggesting that gallbladder neuroendocrine differentiation develops from adenocarcinomas and that both components arise from a single cancer stem cell.

In 1987, Lewin [8] first proposed dividing mixed exocrine-neuroendocrine tumors into three categories: composite tumors, collision tumors, and amphicrine tumors. Fujiyoshi et al. [10] reclassified these tumors by dividing them into six categories: neuroendocrine cells interspersed within carcinomas, carcinoids (NETs) with interspersed nonendocrine cells, composite glandular-neuroendocrine cell carcinomas, collision tumors, amphicrine tumors, and combinations of the previous types. In this study, we reviewed 9 cases of gallbladder MiNENs, including 8 composite tumors and 1 amphicrine carcinoma. To the best of our knowledge, this is the second case in which amphicrine carcinoma has been reported in the gallbladder. Zhang et al. [40] presented the first case of gallbladder amphicrine carcinoma, in which the patient received surgical resection and adjuvant capecitabine chemotherapy with disease-free survival after 8 months of surgery. Regarding composite tumors, the histomorphology revealed that the surface mucosa comprised the adenocarcinoma component, and the neuroendocrine component was in the area of deep invasion, the liver and lymph node metastasis. In the GB-MiNENs patients with histological information on metastases, most of the lymph node metastases, liver infiltration, and distant metastases were composed of a pure neuroendocrine component [14, 18, 21, 23, 29, 34, 37]. On the basis of these findings, the neuroendocrine component appears to be more invasive than the adenocarcinoma component.

The standard therapeutic strategy for GB-NENs has not yet been established. Surgical management remains the first choice for early-stage patients and was also offered to some advanced patients. For Tis and stage I tumors, a cholecystectomy could be enough. Chemotherapy is the first treatment option for advanced patients, and the combination of cisplatin and etoposide has been effective [45]. Furthermore, somatostatin analogs have also been used, with partial success [46].

At present, prognostic factors for NENs are still controversial, and the prognosis depends not only on the stage of the disease but also on the exact histological type [47]. Shi et al. considered that the survival time of patients with GB-MiNENs was similar to that of patients with GB-NECs [48]. Harada et al. [21] concluded that the presence of the neuroendocrine tumor component of MiNENs in the biliary tract defines the prognosis. Moreover, most of the MiNENs cases had a high-grade neuroendocrine component, which has the tendency to invade lymph nodes and adjacent hepatic tissues, resulting in a dismal prognosis even after complete resection [12, 31]. In our study, most tumors were diagnosed at an advanced stage, and all of the patients received surgical resection with a median survival time of 11.5 months. Similarly, one study showed that the median survival time of 20 patients with curatively resected biliary neuroendocrine tumors was 13.7 months [49]. Total analysis combined with GB-MiNENs cases reported in the literature revealed that liver metastasis and tumor stage were significant predictors of OS, and liver metastasis was an independent unfavorable prognostic factor.

The main limitation of the present study was the lack of a statistical power analysis due to the small number of patients. Additionally, some laboratory indicators should be evaluated in future investigations to improve GB-MiNENs prognosis.

## 5. Conclusions

Although there is a low incidence of GB-NENs, our results provide a good picture of the behavior of this rare condition. GB-MiNENs were the most common type in our case series. More importantly, however, the neuroendocrine component of GB-MiNENs follows an aggressive clinical course, as shown in our patients, with higher local invasion and lymph node metastasis than the adenocarcinoma. Liver metastasis was an independent negative prognostic factor for the survival of GB-MiNENs patients in this retrospective study.

## Figures and Tables

**Table 1:** Clinicopathological characteristics of the 13 patients with GB-NENs.

Patient	Sex/age	Presentation	Liver invasion	Lymph node metastasis	Tumor stage	Treatment	Survival (months)
1	M/57	NO	−	+	IIIB (pT3N1M0)	Radical Cho	D (11)
2	F/63	Fever	−	−	IIB (pT2bN0M0)	Radical Cho, Che	D (13)
3	F/70	NO	−	−	IIIA (pT3N0M0)	Radical Cho, Che	D (27)
4	F/53	Backache	+	+	IIIB (pT3N1M0)	Radical Cho	D (5)
5	M/37	NO	−	−	IIIA (pT3N0M0)	Radical Cho, Che	D (12)
6	M/57	Right upper abdominal pain	+	−	IIIA (pT3N0M0)	Radical Cho	D (3)
7	M/64	Backache	−	−	IIB (pT2bN0M0)	Radical Cho, Che	DFS (54)
8	F/75	Right upper abdominal pain	−	−	IIA (pT2aN0M0)	Radical Cho	D (10)
9	F/60	NO	−	−	IIB (pT2bN0M0)	Radical Cho	D (40)
10	F/56	NO	+	+	IVB (pT1N2M0)	Radical Cho, Che	D (15)
11	M/59	Right upper abdominal pain	−	+	IIIB (pT3N1M0)	Radical Cho, Che	D (10)
12	F/58	Right upper abdominal pain	−	−	IB (pT1bN0M0)	Cho	DFS (229)
13	M/35	Right upper abdominal pain	−	−	IB (pT1bN0M0)	Radical Cho	DFS (6)

pTNM: pathological tumor-node-metastasis; Che: chemotherapy; Cho: cholecystectomy; DFS: disease-free survival; D: death.

**Table 2:** Pathology of 13 patients with GB-NENs.

Patient	Site	Morphology	Tumor size	Mitotic index (/10 HPFs)	Neuroendocrine tumor grade	Neuroendocrine neoplasm histologic subtype	Adenocarcinoma component
1	Body	Infiltrative	2	30	MiNENs-NEC	Large cell type	Moderately differentiated
2	Fundus	Infiltrative	3.5	20	MiNENs-NEC	Large cell type	Moderately differentiated
3	Neck	Infiltrative	2.5	20	MiNENs-NEC	Small cell type	Well differentiated
4	Neck	Protruding	4	50	MiNENs-NEC	Large cell type	Well differentiated
5	Body	Infiltrative	5.5	40	MiNENs-NEC	Small cell type	Well differentiated
6	Fundus	Protruding	5	20	MiNENs-NEC	Large cell type	Moderately differentiated
7	Body	Infiltrative	2	30	MiNENs-NEC	Small cell type	Well differentiated
8	Fundus	Infiltrative	7	10	MiNENs-NET G2	Neuroendocrine tumor	Well differentiated
9	Neck	Protruding	3	40	Amphicrine carcinoma	Amphicrine carcinoma	
10	Fundus	Protruding	2	30	NEC	Small cell type	Absent
11	Fundus	Protruding	4	40	NEC	Large cell type	Absent
12	Fundus	Protruding	0.6	1	NET-G1	Neuroendocrine tumor	Absent
13	Fundus	Protruding	0.6	1	NET-G1	Neuroendocrine tumor	Absent

**Table 3:** Immunohistochemistry for GB-NENs.

	Syn	CgA	Ki-67	CD56	CK7	CK19	Cytokeratin
Case 1	+	+	40%	+	NP	+Focal	+
Case 2	+	+	85%	+	+	+Focal	+
Case 3	+	+Focal	50%	−	−	NP	+
Case 4	+	−	70%	−	NP	NP	+
Case 5	+	+	55%	+	NP	NP	+
Case 6	+	+	80%	−	NP	NP	+
Case 7	+	+	65%	+	NP	+	+
Case 8	+	+	10%	+	+	NP	+
Case 9	+	+	60%	+	+	+Focal	+
Case 10	+	+	50%	+	NP	NP	+
Case 11	+	+	60%	+	+	NP	+
Case 12	+	+	2%	_	NP	NP	+
Case 13	+	+	1%	NP	NP	−	NP

Syn: synaptophysin; CgA: chromogranin A; NP: not performed.

**Table 4:** Characteristics of 41 previously reported patients with GB-MiNENs, including our current 9 patients.

Total number of patients	41
Sex	Male: 12 (12/41, 29.2%)
	Female: 29 (29/41, 70.7%)
	Female-to-male ratio: 2.4 : 1
Median age	60 years (age range: 34-85 years)
Primary tumor location	Gallbladder
Tumor site	Fundus: 16 (16/29, 55.2%)
	Body: 5 (7/29, 24.1%)
	Neck: 4 (4/29, 13.8%)
	Fundus and body: 2 (2/29, 6.9%)
Metastatic site	Lymph nodes: 15 (15/41, 36.6%)
	Liver: 17 (17/41, 41.5%)
	Duodenum: 1 (1/41, 2.4%)
	Bone: 1 (1/41, 2.4%)
Presenting symptoms	Abdominal pain: 22 (22/35, 62.9%)
	Fever: 2 (2/35, 5.7%)
	Back pain: 3 (3/35, 8.5%)
	Asymptomatic: 7 (7/35, 20.0%)
	Anorexia: 1 (1/35, 2.9%)
Morphology	Protruding: 27 (27/36, 75.0%)
	Infiltrative: 9 (9/36, 25.0%)
Histopathology of the neuroendocrine component	SCC: 9 (9/41, 22.0%)
	LCNEC: 19 (19/41, 46.3%)
	SCC and LCNEC: 1 (1/41, 2.4%)
	NEC: 6 (6/41, 14.6%)
	NET: G2, 4 (4/41, 9.8%)
	Amphicrine carcinoma: 2 (2/41, 4.9%)
Outcome	Median survival: 11.5 months (range: 2-40 months)
	Median disease-free survival: 12 months (range: 2-48 months)

**Table 5:** The Kaplan-Meier analysis of the overall survival of GB-MiNENs patients.

Variable	Group	Event	*P* value
Age (years)	<60	14	0.300
	≥60	16	
Sex	Male	10	0.238
	Female	20	
Diameter	<4.9	21	0.695
	≥4.9	7	
	NA	2	
Neuroendocrine component	NET	3	0.709
	NEC	27	
Positive LN	Yes	8	0.535
	No	22	
Liver metastasis	Yes	10	0.013^∗^
	No	20	
Tumor stage	I + II	13	0.043^∗^
	III + IV	15	
	NA	2	
Adjuvant chemotherapy	Yes	15	0.916
	No	15	

^∗^ *P* < 0.05.

**Table 6:** The Cox regression and univariate and multivariate analyses of GB-MiNENs.

Parameter	Univariate analysis		Multivariate analysis	
	HR (95% CI)	*P* value	HR (95% CI)	*P* value
Age	0.983 (0.945-1.023)	0.394		
Sex (male vs. female)	0.576 (0.202-1.641)	0.301		
Tumor diameter	1.295 (0.904-1.855)	0.159	1.525 (0.969-2.402)	0.068
Grade of the neuroendocrine component (NET vs. NEC)	1.531 (0.199-11.754)	0.682		
Lymph node metastasis (negative vs. positive)	1.754 (0.547-5.628)	0.345		
Liver metastasis (negative vs. positive)	4.364 (1.503-12.673)	0.007^∗^	4.262 (1.066-17.044)	0.040^∗^
Tumor stage (stages I + II vs. stages III + IV)	3.028 (0.961-9.545)	0.059	1.341 (0.322-5.582)	0.686
Adjuvant chemotherapy	1.020 (0.355-2.933)	0.971		

Variables with *P* < 0.2 in the univariate analysis were included in the multivariate analysis. ^∗^*P* < 0.05.

## Data Availability

The data used to support the findings of this study are included within the article.
